# Operations and structures derived from non-associative MV-algebras

**DOI:** 10.1007/s00500-018-3309-4

**Published:** 2018-06-15

**Authors:** Ivan Chajda, Radomir Halaš, Helmut Länger

**Affiliations:** 1 Department of Algebra and Geometry, Faculty of Science, Palacký University Olomouc, 17. listopadu 12, 771 46 Olomouc, Czech Republic; 2 Institute of Discrete Mathematics and Geometry, Faculty of Mathematics and Geoinformation, TU Wien, Wiedner Hauptstraße 8-10, 1040 Vienna, Austria

**Keywords:** MV-algebra, Non-associative MV-algebra, Implication, Congruence conditions, Sheffer operation

## Abstract

The so-called non-associative MV-algebras were introduced recently by the first author and J. Kühr in order to have an appropriate tool for certain logics used in expert systems where associativity of the binary operation is excluded, see, e.g., Botur and Halaš (Arch Math Log 48:243–255, 2009). Since implication is an important logical connective in practically every propositional logic, in the present paper we investigate the implication reducts of non-associative MV-algebras. We also determine their structures based on the underlying posets. The natural question when a poset with the greatest element equipped with sectional switching involutions can be organized into an implication NMV-algebra is solved. Moreover, congruence properties of the variety of implication NMV-algebras with, respectively, without zero are investigated. Analogously to classical propositional logic, we introduce a certain kind of Sheffer operation and we obtain a one-to-one correspondence between NMV-algebras and certain algebras built up by a Sheffer-like operation together with a unary operation.

## Introduction

The role of MV-algebras introduced in Chang (1958) for multiple-valued reasoning is well known, see, e.g., the monograph (Cignoli et al. 2000). As shown by Botur and Halaš (2009), in some problems concerning expert systems in particular or in artificial intelligence in general, associativity of the binary operation of an MV-algebra can produce serious problems, see also, e.g., Chajda and Länger (2017) for motivation. This was the reason why the so-called non-associative MV-algebras were introduced and studied in Chajda and Kühr (2007) and Chajda and Länger (2017). Since MV-algebras form an algebraic semantics of fuzzy logics and because implication is the most fundamental logical connective, some attempts were made to describe so-called implication reducts of MV-algebras. Such reducts were investigated for MV-algebras in Chajda et al. (2004a) under the name weak implication algebras. However, it turns out that these implication reducts are in fact BCK-algebras and the investigations in Chajda et al. (2004a) provide a new axiomatization of BCK-algebras which is very similar to that derived by Abbott (1967).

Our first goal is to derive an implication algebra (or implication reduct) of the above-mentioned non-associative MV-algebras. The main difference to other implication reducts, e.g., for Boolean algebras in Abbott (1967), for MV-algebras in Chajda et al. (2004a) or for orthomodular lattices or ortholattices in Abbott (1976), Chajda et al. (2001), Chajda et al. (2004b) and Chajda et al. (2008) is that non-associative MV-algebras do not have a lattice as their underlying structure. Namely, their underlying structure is only a bounded poset equipped with involutions on principal order filters. Hence, our attempt is rather exceptional, but it turns out that it works well and the results are fully comparable with that corresponding to lattice structures.

The second motivation is the fact that the so-called Sheffer operation alias Sheffer stroke was studied by several authors in Boolean algebras (Sheffer 1913), MV-algebras, basic algebras (Oner and Senturk 2017), orthomodular lattices and ortholattices (Chajda 2005), but to our knowledge not on structures which are posets only. In the present paper, we show that we can also introduce and investigate a Sheffer stroke operation in non-associative MV-algebras.

## Implication NMV-algebras

For the reader’s convenience, we repeat the definition of our basic concept.

### Definition 2.1

A *non-associative MV-algebra* (*NMV-algebra*, for short) is an algebra $${\mathbf A}=(A,\oplus ,$$$$\lnot ,0)$$ of type (2, 1, 0) satisfying the identities

1 $$\begin{aligned}&x\oplus y \approx y\oplus x, \end{aligned}$$

2 $$\begin{aligned}&x\oplus 0 \approx x, \end{aligned}$$

3 $$\begin{aligned}&x\oplus 1 \approx 1, \end{aligned}$$

4 $$\begin{aligned}&\lnot (\lnot x) \approx x, \end{aligned}$$

5 $$\begin{aligned}&\lnot (\lnot x\oplus y)\oplus y \approx \lnot (\lnot y\oplus x)\oplus x, \end{aligned}$$

6 $$\begin{aligned}&\lnot x\oplus (x\oplus y) \approx 1, \end{aligned}$$

7 $$\begin{aligned}&x\oplus (\lnot (\lnot (\lnot (x\oplus y)\oplus y)\oplus z)\oplus z) \approx 1, \end{aligned}$$

where 1 denotes the algebraic constant $\lnot 0$. Identity (5) is called the *Łukasiewicz axiom*. We define

$$\begin{aligned} x\le y\text { if and only if }\lnot x\oplus y=1 \end{aligned}$$

($$x,y\in A$$).

As shown in Chajda and Kühr (2007), $(A,\le )$ is a poset with the least element 0 and the greatest element 1 which we call the *poset induced* by $\mathbf A$.

### Example 2.2

**Fig. 1:**
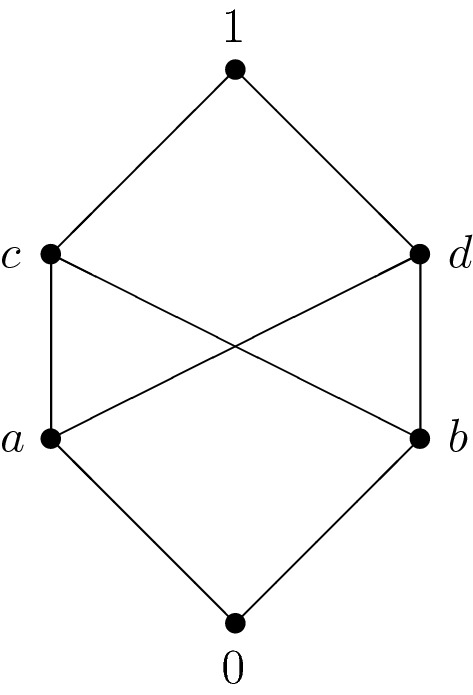
Hasse diagram of the poset induced by an NMV-algebra

The algebra $\mathbf A=(A,\oplus ,\lnot ,0)$ of type (2, 1, 0) defined by $A=\{0,a,b,c,d,1\}$,

$$\begin{aligned} \begin{array}{c|cccccc} \oplus &{} 0 &{} \quad a &{} \quad b &{} \quad c &{} \quad d &{} \quad 1 \\ \hline 0 &{} 0 &{} \quad a &{} \quad b &{} \quad c &{} \quad d &{} \quad 1 \\ a &{} a &{} \quad d &{} \quad c &{} \quad c &{} \quad 1 &{} \quad 1 \\ b &{} b &{} \quad c &{} \quad d &{} \quad 1 &{} \quad d &{} \quad 1 \\ c &{} c &{} \quad c &{} \quad 1 &{} \quad 1 &{} \quad 1 &{} \quad 1 \\ d &{} d &{} \quad 1 &{} \quad d &{} \quad 1 &{} \quad 1 &{} \quad 1 \\ 1 &{} 1 &{} \quad 1 &{} \quad 1 &{} \quad 1 &{} \quad 1 &{} \quad 1 \end{array} \quad \text { and }\quad \begin{array}{r|cccccc} x &{} 0 &{} \quad a &{} \quad b &{} \quad c &{} \quad d &{} \quad 1 \\ \hline \lnot x &{} 1 &{} \quad d &{} \quad c &{} \quad b &{} \quad a &{} \quad 0 \end{array} \end{aligned}$$

is an NMV-algebra whose induced poset has the Hasse diagram depicted in Fig. 1.

One can immediately see that the above poset is not a lattice. Moreover, $\mathbf A$ is not an MV-algebra since the operation $\oplus$ is not associative:

$$\begin{aligned} (a\oplus a)\oplus b=d\oplus b=d\ne c=a\oplus c=a\oplus (a\oplus b). \end{aligned}$$

### Definition 2.3

An *implication NMV-algebra* is a non-empty groupoid $\mathbf A=(A,\rightarrow )$ satisfying the identities

8 $$\begin{aligned}&x\rightarrow x \approx y\rightarrow y, \end{aligned}$$

9 $$\begin{aligned}&x\rightarrow 1 \approx 1, \end{aligned}$$

10 $$\begin{aligned}&1\rightarrow x \approx x, \end{aligned}$$

11 $$\begin{aligned}&(x\rightarrow y)\rightarrow y \approx (y\rightarrow x)\rightarrow x, \end{aligned}$$

12 $$\begin{aligned}&x\rightarrow (y\rightarrow x) \approx 1, \end{aligned}$$

13 $$\begin{aligned}&x\rightarrow ((((x\rightarrow y)\rightarrow y)\rightarrow z)\rightarrow z) \approx 1, \end{aligned}$$

where 1 denotes the algebraic constant $x\rightarrow x$. We define

$$\begin{aligned} x\le y\text { if and only if }x\rightarrow y=1 \end{aligned}$$

($$x,y\in A$$) and put

$$\begin{aligned} x\sqcup y:=(x\rightarrow y)\rightarrow y \end{aligned}$$

for all $x,y\in A$. An *implication NMV-algebra with 0* is an algebra $\mathbf A=(A,\rightarrow ,0)$ of type (2, 0) satisfying identities (8)–(13) as well as the identity

14 $$\begin{aligned} 0\rightarrow x\approx 1. \end{aligned}$$

We put

$$\begin{aligned}&\lnot x := x\rightarrow 0, \\&x\sqcap y := \lnot (\lnot x\sqcup \lnot y) \end{aligned}$$

for all $x,y\in A$ and call $\lnot$ the *negation*.

### Lemma 2.4

For an implication NMV-algebra $(A,\rightarrow )$, the relation $\le$ defined above is a partial order relation on *A* with the greatest element 1.

### Proof

Let $a,b,c\in A$. Then, $a\le a$ according to (8). If $a\le b$ and $b\le a$, then

$$\begin{aligned} a= & {} 1\rightarrow a=(b\rightarrow a)\rightarrow a=(a\rightarrow b)\rightarrow b\\= & {} 1\rightarrow b=b \end{aligned}$$

according to (10) and (11). If $a\le b$ and $b\le c$, then

$$\begin{aligned} a\rightarrow c= & {} a\rightarrow (1\rightarrow c)=a\rightarrow ((b\rightarrow c)\rightarrow c)\\= & {} a\rightarrow (((1\rightarrow b)\rightarrow c)\rightarrow c)\\= & {} a\rightarrow ((((a\rightarrow b)\rightarrow b)\rightarrow c)\rightarrow c)=1 \end{aligned}$$

according to (10) and (13), i.e., $a\le c$. Finally, $a\le 1$ according to (9). $\square$

The partial order relation $\le$ on *A* will be called the *induced order* of $(A,\rightarrow )$.

Let $(P,\le )$ be a poset with the smallest element *p* and the greatest element *q* and $f:P\rightarrow P$. Then, *f* is called

- an *involution* if $f(f(x))=x$ for all $x\in P$,
- *antitone* if $x,y\in P$ and $x\le y$ together imply $f(y)\le f(x)$,
- *switching* if $f(p)=q$ and $f(q)=p$.

### Lemma 2.5

In every implication NMV-algebra $(A,\rightarrow ,0)$ with 0 the negation is a switching involution on $(A,\le )$.

### Proof

We have

$$\begin{aligned}&\lnot 0 \approx 0\rightarrow 0\approx 1\text { according to (8)}, \\&\lnot 1 \approx 1\rightarrow 0\approx 0\text { according to (10)}, \\&\lnot (\lnot x) \approx (x\rightarrow 0)\rightarrow 0\approx (0\rightarrow x)\rightarrow x\approx 1\rightarrow x\approx x \\&\quad \text { according to (10), (11) and (14)}. \end{aligned}$$

$$\square $$

Analogously, as it was done for MV-algebras in Chajda et al. (2004a), we can introduce the binary operation $\rightarrow$ in NMV-algebras which can be interpreted as the logical connective implication within the corresponding logic. The following theorem justifies the name implication NMV-algebra introduced in Definition 2.3.

### Theorem 2.6

Let $\mathbf A=(A,\oplus ,\lnot ,0)$ be an NMV-algebra and define

$$\begin{aligned} x\rightarrow y:=\lnot x\oplus y \end{aligned}$$

for all $x,y\in A$. Then, $(A,\rightarrow ,0)$ is an implication NMV-algebra with 0 which we call the *implication NMV-algebra with 0 induced* by $\mathbf A$.

### Proof

(8) follows from (2) and (6), (9) from (3), (10) from (1), (2) and (4), (11) from (5), (12) from (1) and (6), (13) from (7) and (14) from (1) and (3). $\square$

### Example 2.7

The implication NMV-algebra $\mathbf B:=(A,\rightarrow ,0)$ with 0 induced by the NMV-algebra $\mathbf A$ from Example 2.2 is given by the operation table

$$\begin{aligned} \begin{array}{c|cccccc} \rightarrow &{} 0 &{} \quad a &{} \quad b &{} \quad c &{} \quad d &{} \quad 1 \\ \hline 0 &{} 1 &{} \quad 1 &{} \quad 1 &{} \quad 1 &{} \quad 1 &{} \quad 1 \\ a &{} d &{} \quad 1 &{} \quad d &{} \quad 1 &{} \quad 1 &{} \quad 1 \\ b &{} \quad c &{} \quad c &{} \quad 1 &{} \quad 1 &{} \quad 1 &{} \quad 1 \\ c &{} \quad b &{} \quad c &{} \quad d &{} \quad 1 &{} \quad d &{} \quad 1 \\ d &{} a &{} \quad d &{} \quad c &{} \quad c &{} \quad 1 &{} \quad 1 \\ 1 &{} 0 &{} \quad a &{} \quad b &{} \quad c &{} \quad d &{} \quad 1 \end{array} \end{aligned}$$

**Fig. 2:**
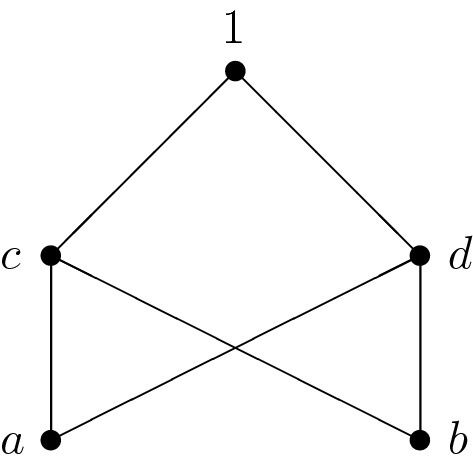
Hasse diagram of the poset induced by an implication NMV-algebra

Since $C:=\{a,b,c,d,1\}$ is a subuniverse of the implication NMV-algebra $(B,\rightarrow )$, the groupoid $(C,\rightarrow )$ is an implication NMV-algebra, too. But there exists no $x\in \{a,b,c,d,1\}$ such that $(C,\rightarrow ,x)$ is an implication NMV-algebra with 0. The Hasse diagram of the poset induced by $(C,\rightarrow )$ is visualized in Fig. 2.

### Theorem 2.8

Let $\mathbf A=(A,\rightarrow ,0)$ be an implication NMV-algebra with 0 and define a binary operation $\oplus$ on *A* by

$$\begin{aligned} x\oplus y:=\lnot x\rightarrow y \end{aligned}$$

for all $x,y\in A$. Then, $(A,\oplus ,\lnot ,0)$ is an NMV-algebra if and only if $\mathbf A$ satisfies the identity

15 $$\begin{aligned} x\rightarrow y\approx \lnot y\rightarrow \lnot x. \end{aligned}$$

### Proof

We have

$$\begin{aligned} x\rightarrow y\approx \lnot (\lnot x)\rightarrow y\approx \lnot x\oplus y \end{aligned}$$

according to Lemma 2.5. If $(A,\oplus ,\lnot ,0)$ is an NMV-algebra, then

$$\begin{aligned} x\rightarrow y\approx \lnot x\oplus y\approx y\oplus \lnot x\approx \lnot y\rightarrow \lnot x \end{aligned}$$

according to (1). If, conversely, $\mathbf A$ satisfies identity (15), then

1. $x\oplus y\approx \lnot x\rightarrow y\approx \lnot y\rightarrow \lnot (\lnot x)\approx \lnot y\rightarrow x\approx y\oplus x$ according to Lemma 2.5,
2. $x\oplus 0\approx \lnot x\rightarrow 0\approx \lnot (\lnot x)\approx x$ according to Lemma 2.5,
3. $$x\oplus 1\approx \lnot x\rightarrow 1\approx 1$$ according to (9),
4. was proved just before,
5. $$\lnot (\lnot x\oplus y)\oplus y\approx (x\rightarrow y)\rightarrow y\approx (y\rightarrow x)\rightarrow x\approx \lnot (\lnot y\oplus x)\oplus x$$ according to (11),
6. $\lnot x\oplus (x\oplus y)\approx \lnot x\oplus (y\oplus x)\approx x\rightarrow (\lnot y\rightarrow x)\approx 1$ according to (1) and (12),
7. $$x\oplus (\lnot (\lnot (\lnot (x\oplus y)\oplus y)\oplus z)\oplus z)\approx \lnot x\rightarrow ((((\lnot x\rightarrow y)\rightarrow y)\rightarrow z)\rightarrow z)\approx 1$$ according to (13). $$\square $$

Recall the following concept (see, e.g., Chajda and Länger 2011 or Ježek and Quackenbush 1990):

### Definition 2.9

A *directoid* is a groupoid $(A,\sqcup )$ satisfying the identities

$$\begin{aligned}&x\sqcup x \approx x, \\&x\sqcup y \approx y\sqcup x, \\&x\sqcup ((x\sqcup y)\sqcup z) \approx (x\sqcup y)\sqcup z. \end{aligned}$$

A *directoid with 1* is an algebra $(A,\sqcup ,1)$ of type (2, 0) such that $(A,\sqcup )$ is a directoid and the identity $x\sqcup 1\approx 1$ is satisfied.

The concept of a directoid was introduced under the name *commutative directoid with 1* by Ježek and Quackenbush (1990).

### Definition 2.10

A *poset*$(A,\le )$ is called *directed* if for any $x,y\in A$ we have $U(x,y)\ne \emptyset$ where $U(x,y):=\{z\in A\mid x,y\le z\}$.

For the following result, see, e.g., Chajda and Länger (2011) or Ježek and Quackenbush (1990).

### Proposition 2.11

If $\mathbf A=(A,\sqcup )$ is a directoid and we define a binary relation $\le$ on *A* by

$$\begin{aligned} x\le y\text { if and only if }x\sqcup y=y \end{aligned}$$

($x,y\in A$), then $\mathbb P(\mathbf A):=(A,\le )$ is a directed poset satisfying $x,y\le x\sqcup y$. The partial order relation on *A* just defined will be called the *induced order* of the directoid $(A,\sqcup )$. Conversely, if $\mathbf P=(A,\le )$ is a directed poset and we define

$$\begin{aligned}&x\sqcup y:=\max (x,y)\text { if }x\text { and }y\text { are comparable and }x\sqcup y\\&\quad =y\sqcup x\in U(x,y)\text { otherwise} \end{aligned}$$

($x,y\in A$), then $\mathbb D(\mathbf P):=(A,\sqcup )$ is a directoid. (In general, $\mathbb D(\mathbf P)$ is not uniquely determined by $\mathbf P$.) We have $\mathbb P(\mathbb D(\mathbf P))=\mathbf P$ for every directed poset $\mathbf P$.

In the following, if $(A,\le )$ is a poset and *B* a subset of *A*, then the restriction of $\le$ to *B* will again be denoted by the same symbol $\le$.

### Definition 2.12

A *directoid with 1 and sectionally switching involutions* is an ordered quadruple $(A,\sqcup ,1,(f_a;a\in A))$ such that $(A,\sqcup ,1)$ is a directoid with 1 and for every $a\in A$, $f_a$ is a switching involution on $([a,1],\le )$ where $\le$ is the induced order of $(A,\sqcup )$.

### Theorem 2.13

Let $\mathbf A=(A,\rightarrow )$ be an implication NMV-algebra and for each $a\in A$ define a mapping $f_a:[a,1]\rightarrow [a,1]$ by

$$\begin{aligned} f_a(x):=x\rightarrow a \end{aligned}$$

for all $x\in [a,1]$. Then, $\mathbb D_1(\mathbf A):=(A,\sqcup ,1,(f_a;a\in A))$ where $\sqcup$ denotes the binary operation on *A* introduced in Definition 2.3 is a directoid with 1 and sectionally switching involutions whose induced order coincides with the induced order of $(A,\rightarrow )$.

### Proof

Let $$a,b,c\in A$$. Then,

$$\begin{aligned}&a\sqcup a = (a\rightarrow a)\\&\quad \rightarrow a=1\rightarrow a=a\text { according to (8) and (10)}, \\&a\sqcup b = (a\rightarrow b)\rightarrow b=(b\rightarrow a)\\&\quad \rightarrow a=b\sqcup a\text { according to (11)}, \\&a\sqcup ((a\sqcup b)\sqcup c) = (a\rightarrow ((((a\rightarrow b)\rightarrow b)\\&\quad \rightarrow c)\rightarrow c))\rightarrow \\&\quad \rightarrow ((((a\rightarrow b)\rightarrow b)\rightarrow c)\rightarrow c)=1\\&\quad \rightarrow ((((a\rightarrow b)\rightarrow b)\rightarrow c)\rightarrow c) \\&\quad = (((a\rightarrow b)\rightarrow b)\rightarrow c)\rightarrow c\\&\quad =(a\sqcup b)\sqcup c\text { according to (10) and} \\&\qquad \text {(13)}, \\&a\sqcup 1 = (a\rightarrow 1)\rightarrow 1\\&\quad =1\text { according to (9)}, \\&a\sqcup b=b \Rightarrow a\rightarrow b=a\rightarrow (a\sqcup b)\\&\quad =a\rightarrow ((a\rightarrow b)\rightarrow b)= \\&\quad = a\rightarrow ((b\rightarrow a)\rightarrow a)=1\text { according to (11) and (12)}, \\&a\rightarrow b=1 \Rightarrow a\sqcup b=(a\rightarrow b)\rightarrow b=1\\&\quad \rightarrow b=b\text { according to (10)}. \end{aligned}$$

If $$b\in [a,1]$$, then

$$\begin{aligned}&f_a(b) = b\rightarrow a\ge a\text { according to (12)}, \\&f_a(f_a(b)) = (b\rightarrow a)\rightarrow a=(a\rightarrow b)\rightarrow b=1\rightarrow b\\&\quad =b\text { according to (10) and (11)}, \\&f_a(a) = a\rightarrow a=1\text { according to (8)}, \\&f_a(1) = 1\rightarrow a=a\text { according to (10)}. \end{aligned}$$

$$\square $$

For an implication NMV-algebra $\mathbf A=(A,\rightarrow )$, $\mathbb D_1(\mathbf A)=(A,\sqcup ,1,(f_a;a\in A))$ will be called the *induced directoid*.

### Example 2.14

For the implication NMV-algebra $$\mathbf B$$ from Example 2.7, $$\mathbb D_1(\mathbf B)=(A,\sqcup ,$$$$1,(f_a;a\in A))$$ is given by the tables

$$\begin{aligned} \begin{array}{c|cccccc} \sqcup &{} 0 &{} \quad a &{} \quad b &{} \quad c &{} \quad d &{} \quad 1 \\ \hline 0 &{} 0 &{} \quad a &{} \quad b &{} \quad c &{} \quad d &{} \quad 1 \\ a &{} a &{} \quad a &{} \quad c &{} \quad c &{} \quad d &{} \quad 1 \\ b &{} b &{} \quad c &{} \quad b &{} \quad c &{} \quad d &{} \quad 1 \\ c &{} c &{} \quad c &{} \quad c &{} \quad c &{} \quad 1 &{} \quad 1 \\ d &{} d &{} \quad d &{} \quad d &{} \quad 1 &{} \quad d &{} \quad 1 \\ 1 &{} 1 &{} \quad 1 &{} \quad 1 &{} \quad 1 &{} \quad 1 &{} \quad 1 \end{array} \quad \text { and }\quad \begin{array}{c|cccccc} x &{} 0 &{} \quad a &{} \quad b &{} \quad c &{} \quad d &{} \quad 1 \\ \hline f_0(x) &{} 1 &{} \quad d &{} \quad c &{} \quad b &{} \quad a &{} \quad 0 \\ f_a(x) &{} &{} \quad 1 &{} &{} \quad c &{} \quad d &{} \quad a \\ f_b(x) &{} &{} &{} \quad 1 &{} \quad d &{} \quad c &{} \quad b \\ f_c(x) &{} &{} &{} &{} \quad 1 &{} &{} \quad c \\ f_d(x) &{} &{} &{} &{} &{} \quad 1 &{} \quad d \\ f_1(x) &{} &{} &{} &{} &{} &{} \quad 1 \end{array} \end{aligned}$$

### Theorem 2.15

Let $\mathbf D=(A,\sqcup ,1,(f_a;a\in A))$ be a directoid with 1 and sectionally switching involutions and define a binary operation $\rightarrow$ on *A* by

$$\begin{aligned} x\rightarrow y:=f_y(x\sqcup y) \end{aligned}$$

for all $x,y\in A$. Then, $\mathbb I(\mathbf D):=(A,\rightarrow )$ is an implication NMV-algebra.

### Proof

We have $(x\rightarrow y)\rightarrow y\approx f_y(f_y(x\sqcup y)\sqcup y)\approx f_y(f_y(x\sqcup y))\approx x\sqcup y$. Now the following identities are satisfied:

- (8) $$x\rightarrow x\approx f_x(x\sqcup x)\approx f_x(x)\approx 1\approx f_y(y)\approx f_y(y\sqcup y)\approx y\rightarrow y$$,
- (9) $$x\rightarrow 1\approx f_1(x\sqcup 1)\approx f_1(1)\approx 1$$,
- (10) $$1\rightarrow x\approx f_x(1\sqcup x)\approx f_x(1)\approx x$$,
- (11) $$(x\rightarrow y)\rightarrow y\approx x\sqcup y\approx y\sqcup x\approx (y\rightarrow x)\rightarrow x$$,
- (12) $$x\rightarrow (y\rightarrow x)\approx f_{f_x(y\sqcup x)}(x\sqcup f_x(y\sqcup x))\approx f_{f_x(y\sqcup x)}(f_x(y\sqcup x))\approx 1$$,
- (13) $$x\rightarrow ((((x\rightarrow y)\rightarrow y)\rightarrow z)\rightarrow z)\approx f_{(x\sqcup y)\sqcup z}(x\sqcup ((x\sqcup y)\sqcup z))\approx f_{(x\sqcup y)\sqcup z}((x\sqcup y)\sqcup z)\approx 1$$. $$\square $$

For a directoid $\mathbf D=(A,\sqcup ,1,(f_a;a\in A))$ with 1 and sectionally switching involutions, $\mathbb I(\mathbf D)=(A,\rightarrow )$ will be referred to as the *induced implication NMV-algebra*.

We show that the correspondence between induced directoids and induced implication NMV-algebras is one to one.

### Theorem 2.16

(i) Let $\mathbf A=(A,\rightarrow )$ be an implication NMV-algebra. Then, $\mathbb I(\mathbb D_1(\mathbf A))=\mathbf A$.
(ii) Let $\mathbf D=(A,\sqcup ,1,(f_a;a\in A))$ be a directoid with 1 and sectionally switching involutions. Then, $\mathbb D_1(\mathbb I(\mathbf D))=\mathbf D$.

### Proof

(i) If $\mathbb D_1(\mathbf A)=(A,\sqcup ,1,(f_a;a\in A))$, $\mathbb I(\mathbb D_1(\mathbf A))=(A,\rightarrow ')$ and $a,b\in A$, then
  $$\begin{aligned} a\rightarrow 'b= & {} f_b(a\sqcup b)=(a\sqcup b)\rightarrow b\\= & {} ((a\rightarrow b)\rightarrow b)\rightarrow b= \\= & {} (b\rightarrow (a\rightarrow b))\rightarrow (a\rightarrow b)=1\rightarrow (a\rightarrow b)\\= & {} a\rightarrow b \end{aligned}$$
  according to (10), (11) and (12).
(ii) If $\mathbb I(\mathbf D)=(A,\rightarrow )$, $\mathbb D_1(\mathbb I(\mathbf D))=(A,\sqcup ',1',(g_a;a\in A))$ and $a,b\in A$, then
  $$\begin{aligned} a\sqcup 'b= & {} (a\rightarrow b)\rightarrow b=f_b(f_b(a\sqcup b)\sqcup b) \\= & {} f_b(f_b(a\sqcup b))=a\sqcup b,\\ 1'= & {} a\rightarrow a=f_a(a\sqcup a)=f_a(a)=1, \\ b\in [a,1]\Rightarrow & {} g_a(b)=b\rightarrow a=f_a(b\sqcup a)=f_a(b). \end{aligned}$$

$$\square $$

Next we investigate when the sections of an implication NMV-algebra are NMV-algebras again.

### Theorem 2.17

Let $$\mathbf A=(A,\rightarrow )$$ be an implication NMV-algebra, $$(A,\sqcup ,1,(f_b;$$$$b\in A))$$ its induced directoid, $$a\in A$$, assume

16 $$\begin{aligned} x\rightarrow y=f_a(y)\rightarrow f_a(x) \end{aligned}$$

for all $x,y\in [a,1]$ and put

$$\begin{aligned}&x\oplus _ay := f_a(x)\rightarrow y, \\&\lnot _ax := f_a(x) \end{aligned}$$

for all $x,y\in [a,1]$. Then, $([a,1],\oplus _a,\lnot _a,a)$ is an NMV-algebra.

### Proof

Although some of the following calculations were already done in previous parts of the paper, these calculations were not done in connection with the operations $\oplus _a$ and $\lnot _a$. Hence, for the reader’s convenience, we present the detailed calculations. Let $b,c,d\in [a,1]$. Then,

$$\begin{aligned}&b\oplus _ac = f_a(b)\rightarrow c\ge c\ge a\text { according to (12)}, \\&\lnot _ab = f_a(b)\ge a, \\&\lnot _ab\oplus _ac = f_a(f_a(b))\rightarrow c=b\rightarrow c, \\&b\oplus _ac = f_a(b)\rightarrow c=\lnot _ab\rightarrow c, \end{aligned}$$

- (1) $$b\oplus _ac=f_a(b)\rightarrow c=f_a(c)\rightarrow f_a(f_a(b))=f_a(c)\rightarrow b=c\oplus _ab$$,
- (2) $$b\oplus _aa=f_a(b)\rightarrow a=f_a(a)\rightarrow f_a(f_a(b))=1\rightarrow b=b$$ according to (10),
- (3) $$b\oplus _a1=f_a(b)\rightarrow 1=1$$ according to (9),
- (4) $$\lnot _a(\lnot _ab)=f_a(f_a(b))=b$$,
- (5) $$\lnot _a(\lnot _ab\oplus _ac)\oplus _ac=(b\rightarrow c)\rightarrow c=(c\rightarrow b)\rightarrow b=\lnot _a(\lnot _ac\oplus _ab)\oplus _ab$$ according to (11),
- (6) $$\lnot _ab\oplus _a(b\oplus _ac)=f_a(f_a(b))\rightarrow (f_a(b)\rightarrow c)=b\rightarrow (f_a(c)\rightarrow f_a(f_a(b)))=b\rightarrow (f_a(c)\rightarrow b)=1$$ according to (12),
- (7) $$b\oplus _a(\lnot _a(\lnot _a(\lnot _a(b\oplus _ac)\oplus _ac)\oplus _ad)\oplus _ad)=\lnot _ab\rightarrow ((((\lnot _ab\rightarrow c)\rightarrow c)\rightarrow d)\rightarrow d)=1$$ according to (13).

$$\square $$

Let us note that, contrary to Theorem 2.17, in Theorem 2.15 we do not assume condition (16).

Unfortunately, condition (16) need not be satisfied even in an implication NMV-algebra induced by an NMV-algebra. It is worth noticing that this disadvantage disappears if so-called weak MV-algebras are considered instead of NMV-algebras. These weak MV-algebras were introduced and studied by the Halaš and Plojhar (2008).

### Example 2.18

Consider the implication NMV-algebra $(C,{\rightarrow })$ from Example 2.7. It is easy to see that the element *b* satisfies condition (16). Hence, especially, we have

$$\begin{aligned} c\oplus _bd= & {} (c\rightarrow b)\rightarrow d=d\rightarrow d=1=c\rightarrow c\\= & {} (d\rightarrow b)\rightarrow c=d\oplus _bc. \end{aligned}$$

Contrary to this, the element *a* from Examples 2.7 and 2.14 does not satisfy condition (16), namely

$$\begin{aligned} c\rightarrow d=d\ne c=d\rightarrow c=f_a(d)\rightarrow f_a(c). \end{aligned}$$

This is in accordance with the observation

$$\begin{aligned} c\oplus _ad= & {} (c\rightarrow a)\rightarrow d=c\rightarrow d=d\ne c=d\rightarrow c\\= & {} (d\rightarrow a)\rightarrow c=d\oplus _ac. \end{aligned}$$

If, however, the involution $f_a$ would be given by

$$\begin{aligned} f_a(a)=1,\ f_a(c)=d,\ f_a(d)=c,\ f_a(1)=a, \end{aligned}$$

then (16) would be satisfied also for the element *a* and hence every interval [*x*, 1] for $x\in \{a,b,c,d,1\}$ could be organized into an NMV-algebra which in fact would be an MV-algebra.

## Congruence properties of implication NMV-algebras

The congruence properties of the variety of NMV-algebras were investigated in Chajda and Kühr (2007) and Chajda and Länger (2017). Since implication NMV-algebras form a variety, too, it is natural to ask what congruence properties are satisfied by this variety. One can hardly expect that the variety of implication NMV-algebras satisfies the same congruence properties as the variety of NMV-algebras because in the latter the existence of a zero element plays a fundamental role. On the other hand, we will show that the variety of implication NMV-algebras satisfies similar congruence properties as the varieties of orthoimplication algebras (Abbott 1976), implication MV-algebras (Chajda et al. 2004a) or orthomodular implication algebras (Chajda et al. 2001, 2004b).

Recall that an algebra $\mathbf A=(A,F)$ is called

- *congruence permutable* if $\Theta \circ \Phi =\Phi \circ \Theta$ for all $\Theta ,\Phi \in {{\mathrm{Con}}}\mathbf A$,
- *congruence distributive* if $(\Theta \vee \Phi )\wedge \Psi =(\Theta \wedge \Psi )\vee (\Phi \wedge \Psi )$ for all $\Theta ,\Phi ,\Psi \in {{\mathrm{Con}}}\mathbf A$,
- *arithmetical* if it is both congruence permutable and congruence distributive,
- *congruence regular* if $a\in A$, $\Theta ,\Phi \in {{\mathrm{Con}}}\mathbf A$ and $[a]\Theta =[a]\Phi$ together imply $\Theta =\Phi$,
- *3-permutable* if $\Theta \circ \Phi \circ \Theta =\Phi \circ \Theta \circ \Phi$ for all $\Theta ,\Phi \in {{\mathrm{Con}}}\mathbf A$.

An algebra $\mathbf A$ with an equationally definable constant 1 is called

- *permutable at 1* (or *subtractive*) if $[1](\Theta \circ \Phi )=[1](\Phi \circ \Theta )$ for all $\Theta ,\Phi \in {{\mathrm{Con}}}\mathbf A$,
- *weakly regular* if $\Theta ,\Phi \in {{\mathrm{Con}}}\mathbf A$ and $[1]\Theta =[1]\Phi$ together imply $\Theta =\Phi$.

A variety (with 1 in its similarity type) is said to have the corresponding property if every of its members has this property.

From Chajda et al. (2012), we take the following well-known facts:

Let $\mathcal V$ be a variety. Then, the following hold:

- $\mathcal V$ is congruence permutable if and only if there exists a ternary term *t* in $\mathcal V$ satisfying the identities $t(x,x,y)\approx t(y,x,x)\approx y$ (Theorem 3.1.8),
- If there exists a ternary term *t* in $\mathcal V$ satisfying the identities $t(x,x,y)\approx t(x,y,x)\approx t(y,x,x)\approx x$, then $\mathcal V$ is congruence distributive (Corollary 3.2.4),
- $\mathcal V$ is congruence regular if and only if there exist a positive integer *n* and ternary terms $t_1,\ldots ,t_n$ in $\mathcal V$ such that $t_1(x,y,z)=\cdots =t_n(x,y,z)=z$ is equivalent to $x=y$ (Theorem 6.1.3),
- $\mathcal V$ is 3-permutable if and only if there exist ternary terms $t_1,t_2$ of $\mathcal V$ satisfying the identities $t_1(x,z,z)\approx x$, $t_1(x,x,z)=t_2(x,z,z)$ and $t_2(x,x,z)\approx z$ (Theorem 3.1.18).

A variety $\mathcal V$ with an equationally definable constant 1 is

- permutable at 1 if and only if there exists a binary term *t* in $\mathcal V$ satisfying the identities $t(x,x)\approx 1$ and $t(x,1)\approx x$ (Theorem 6.6.11),
- weakly regular if and only if there exist a positive integer *n* and binary terms $t_1,\ldots ,t_n$ in $\mathcal V$ such that $t_1(x,y)=\cdots =t_n(x,y)=1$ is equivalent to $x=y$ (Theorem 6.4.3).

We are now able to prove

### Lemma 3.1

(i) The variety of groupoids $(A,\rightarrow )$ satisfying identities (8), (10) and (11) is 3-permutable and weakly regular.
(ii) The variety of groupoids $(A,\rightarrow )$ satisfying identities (8) and (10) is permutable at 1.

### Proof

(i) If
  $$\begin{aligned} t_1(x,y,z):= & {} (z\rightarrow y)\rightarrow x, \\ t_2(x,y,z):= & {} (x\rightarrow y)\rightarrow z, \end{aligned}$$
  then
  $$\begin{aligned} t_1(x,z,z)\approx & {} (z\rightarrow z)\rightarrow x\approx 1\rightarrow x\\\approx & {} x\text { according to (8) and (10)}, \\ t_1(x,x,z)\approx & {} (z\rightarrow x)\rightarrow x\approx (x\rightarrow z)\rightarrow z\\\approx & {} t_2(x,z,z)\text { according to (11) and} \\ t_2(x,x,z)\approx & {} (x\rightarrow x)\rightarrow z\approx 1\rightarrow z\\\approx & {} z\text { according to (8) and (10)}. \end{aligned}$$
  If
  $$\begin{aligned} t_1(x,y):= & {} x\rightarrow y, \\ t_2(x,y):= & {} y\rightarrow x, \end{aligned}$$
  then
  $$\begin{aligned}&t_1(x,x) \approx x\rightarrow x\approx 1\text { according to (8)}, \\&t_2(x,x) \approx x\rightarrow x\approx 1\text { according to (8)}, \\&t_1(x,y)=t_2(x,y)=1 \Rightarrow x=1\rightarrow x=(y\rightarrow x) \\&\quad \rightarrow x=(x\rightarrow y)\rightarrow y=1\rightarrow y=y \\&\qquad \text {according to (10) and (11)}. \end{aligned}$$
(ii) If
  $$\begin{aligned} t(x,y):=y\rightarrow x, \end{aligned}$$
  then
  $$\begin{aligned} t(x,x)\approx & {} x\rightarrow x\approx 1\text { according to (8)}, \\ t(x,1)\approx & {} 1\rightarrow x\approx x\text { according to (10)}. \end{aligned}$$

$$\square $$

Since implication NMV-algebras satisfy (8), (10) and (11), we obtain

### Theorem 3.2

The variety of implication NMV-algebras is 3-permutable, permutable at 1 and weakly regular.

In the case of implication NMV-algebras with 0 and antitone negation, we obtain a stronger result.

### Theorem 3.3

The variety of implication NMV-algebras $(A,\rightarrow ,0)$ with 0 satisfying the identity

17 $$\begin{aligned}&((((x\rightarrow y)\rightarrow y)\rightarrow 0)\rightarrow (x\rightarrow 0))\rightarrow (x\rightarrow 0)\nonumber \\&\quad \approx x\rightarrow 0 \end{aligned}$$

is arithmetical and congruence regular.

### Proof

Identity (17) can be rewritten in the form $\lnot (x\sqcup y)\sqcup \lnot x\approx \lnot x$ which is equivalent to the fact that the negation is antitone. If we define $x\sqcap y:=\lnot (\lnot x\sqcup \lnot y)$ for all $x,y\in A$, then the De Morgan laws hold and $\sqcup$ and $\sqcap$ have similar properties as lattice operations do have. If

$$\begin{aligned} t(x,y,z):=((x\rightarrow y)\rightarrow z)\sqcap ((z\rightarrow y)\rightarrow x), \end{aligned}$$

then

$$\begin{aligned} t(x,x,z)\approx & {} ((x\rightarrow x)\rightarrow z)\sqcap ((z\rightarrow x)\rightarrow x) \\\approx & {} (1\rightarrow z)\sqcap (z\sqcup x)\approx z\sqcap (z\sqcup x)\approx z\\&\text {according to (8) and (10)}, \\ t(z,x,x)\approx & {} ((z\rightarrow x)\rightarrow x)\sqcap ((x\rightarrow x)\rightarrow z) \\\approx & {} (z\sqcup x)\sqcap (1\rightarrow z)\approx (z\sqcup x)\sqcap z\approx z\\&\text {according to (8) and (10)}. \end{aligned}$$

If

$$\begin{aligned} t(x,y,z):=((x\sqcup y)\sqcap (y\sqcup z))\sqcap (z\sqcup x), \end{aligned}$$

then

$$\begin{aligned} t(x,x,y)\approx & {} ((x\sqcup x)\sqcap (x\sqcup y))\sqcap (y\sqcup x)\\\approx & {} x\sqcap (y\sqcup x)\approx x, \\ t(x,y,x)\approx & {} ((x\sqcup y)\sqcap (y\sqcup x))\sqcap (x\sqcup x)\\\approx & {} (x\sqcup y)\sqcap x\approx x, \\ t(y,x,x)\approx & {} ((y\sqcup x)\sqcap (x\sqcup x))\sqcap (x\sqcup y)\\\approx & {} x\sqcap (x\sqcup y)\approx x. \end{aligned}$$

If

$$\begin{aligned}&v := (x\rightarrow y)\sqcap (y\rightarrow x), \\&t_1(x,y,z) := v\sqcap z, \\&t_2(x,y,z) := v\rightarrow z, \end{aligned}$$

then

$$\begin{aligned} t_1(x,x,z)\approx & {} ((x\rightarrow x)\sqcap (x\rightarrow x))\sqcap z\approx (1\sqcap 1)\sqcap z\\\approx & {} z\text { according to} \\&\text {(8)}, \\ t_2(x,x,z)\approx & {} ((x\rightarrow x)\sqcap (x\rightarrow x))\rightarrow z\approx (1\sqcap 1)\rightarrow z\\\approx & {} 1\rightarrow z\approx z \\&\text {according to (8)}\text { and (10)}, \\ t_1(x,y,z)= & {} t_2(x,y,z)=z \Rightarrow x\rightarrow y,y\rightarrow x\\\ge & {} v=v\sqcup (v\sqcap z)=v\sqcup z \\= & {} (v\rightarrow z)\rightarrow z=z\rightarrow z=1\\&\text { according to (8) and hence }x\\&\quad \rightarrow y=y\rightarrow x \\= & {} 1\text {whence }x=y. \end{aligned}$$

$$\square $$

## Sheffer stroke NMV-algebras

A binary operation called Sheffer stroke was introduced by Sheffer (1913) in order to have the single operation on a Boolean algebra which generates the clone of all Boolean operations. It has an important application in chip technology since it enables to have all the diods on the chip forming processor in a computer in a uniform manner. This is simpler and cheaper than to produce different diods for disjunction, conjunction and negation. Sheffer operations were also introduced in other algebras which form an algebraic semantic of non-classical logics such as orthomodular lattices, ortholattices (Chajda 2005) or basic algebras (Oner and Senturk 2017). However, all of these algebras have a lattice structure which is not the case for NMV-algebras. Contrary to this, we are able to define a Sheffer operation also for NMV-algebras and their implication reducts.

### Definition 4.1

A *strong Sheffer stroke NMV-algebra* is an algebra (*A*, |, 1) of type (2, 0) satisfying the identities

$$\begin{aligned}&x|y \approx y|x, \\&x|0 \approx 1, \\&(x|1)|1 \approx x, \\&((x|1)|y)|y \approx ((y|1)|x)|x, \\&(x|1)|((x|y)|1) \approx 1, \\&x|(((((x|y)|y)|z)|z)|1) \approx 1, \end{aligned}$$

where 0 denotes the algebraic constant 1|1. The operation | will be called the *strong Sheffer stroke*.

We justify the name strong Sheffer stroke NMV-algebra, respectively, strong Sheffer stroke by the following result.

### Theorem 4.2

Let $\mathbf A=(A,\oplus ,\lnot ,0)$ be an NMV-algebra and put

$$\begin{aligned} x|y:= & {} \lnot x\oplus \lnot y \end{aligned}$$

for all $x,y\in A$. Then, $\mathbb S(\mathbf A):=(A,|,1)$ is a strong Sheffer stroke NMV-algebra.

### Proof

The following identities are satisfied:

$$\begin{aligned}&1|1 \approx \lnot 1\oplus \lnot 1\approx 0, \\&x|y \approx \lnot x\oplus \lnot y\approx \lnot y\oplus \lnot x\approx y|x, \\&x|0 \approx \lnot x\oplus \lnot 0\approx 1, \\&x|1 \approx \lnot x\oplus \lnot 1\approx \lnot x, \\&((x|1)|1 \approx \lnot (\lnot x)\approx x, \\&((x|1)|y)|y \approx \lnot (\lnot \lnot x\oplus \lnot y)\oplus \lnot y \\&\quad \approx \lnot (\lnot \lnot y\oplus \lnot x)\oplus \lnot x\approx ((y|1)|x)|x,\\&(x|1)|((x|y)|1) \approx \lnot \lnot x\oplus \lnot \lnot (\lnot x\oplus \lnot y) \\&\quad \approx \lnot \lnot x\oplus (\lnot x\oplus \lnot y)\approx 1,\\&x|(((((x|y)|y)|z)|z)|1) \approx \lnot x\oplus \lnot \lnot (\lnot (\lnot (\lnot (\lnot x\oplus \lnot y)\\&\quad \oplus \lnot y)\oplus \lnot z)\oplus \lnot z)\approx 1. \end{aligned}$$

$$\square $$

Of course, our main goal is to prove that the strong Sheffer stroke acts on NMV-algebras in an analogous way as the Sheffer stroke does on Boolean algebras, i.e., every operation of an NMV-algebra can be expressed by means of the strong Sheffer operation and the constant 1.

### Theorem 4.3

Let $\mathbf S=(A,|,1)$ be a strong Sheffer stroke NMV-algebra and put

$$\begin{aligned}&x\oplus y := (x|1)|(y|1), \\&\lnot x := x|1, \\&0 := 1|1 \end{aligned}$$

for all $x,y\in A$. Then, $\mathbb A(\mathbf S):=(A,\oplus ,\lnot ,0)$ is an NMV-algebra.

### Proof

The following identities are satisfied:

$$\begin{aligned}&\lnot 0 \approx (1|1)|1\approx 1, \\&\lnot x\oplus y \approx ((x|1)|1)|(y|1)\approx x|(y|1), \end{aligned}$$

- (1) $$x\oplus y\approx (x|1)|(y|1)\approx (y|1)|(x|1)\approx y\oplus x$$,
- (2) $$x\oplus 0\approx (x|1)|(0|1)\approx (x|1)|(1|0)\approx (x|1)|1\approx x$$,
- (3) $$x\oplus 1\approx (x|1)|(1|1)\approx (x|1)|0\approx 1$$,
- (4) $$\lnot (\lnot x)\approx (x|1)|1\approx x$$,
- (5) $$\lnot (\lnot x\oplus y)\oplus y\approx (x|(y|1))|(y|1)\approx (((x|1)|1)|(y|1))|(y|1)\approx (((y|1)|1)|(x|1))|(x|1)\approx (y|(x|1))|(x|1)\approx \lnot (\lnot y\oplus x)\oplus x$$,
- (6) $$\lnot x\oplus (x\oplus y)\approx ((x|1)|1)|(((x|1)|(y|1))|1)\approx 1$$,
- (7) $$x\oplus (\lnot (\lnot (\lnot (x\oplus y)\oplus y)\oplus z)\oplus z) \approx (x|1)|((((((((((((x|1)|(y|1))|1)|1)|(y|1))|1)|1)|(z|1))|1)|1)|(z|1))|1)\approx (x|1)|((((((x|1)|(y|1))|(y|1))|(z|1))|(z|1))|1)\approx 1.$$$$\square $$

We are now able to prove that the correspondence just considered is one to one.

### Theorem 4.4

The above-mentioned correspondence is one to one.

### Proof

If $$\mathbf A=(A,\oplus ,\lnot ,0)$$ is an NMV-algebra, $$\mathbb S(\mathbf A)=(A,|,1)$$ and $$\mathbb A(\mathbb S(\mathbf A))=(A,\oplus _1,$$$$\lnot _1,0_1)$$, then the identities

$$\begin{aligned}&x\oplus _1y \approx (x|1)|(y|1)\approx \lnot (\lnot x\oplus \lnot 1)\oplus \lnot (\lnot y\oplus \lnot 1)\\&\quad \approx x\oplus y, \\&\lnot _1x \approx x|1\approx \lnot x\oplus \lnot 1\approx \lnot x, \\&0_1 \approx 1|1\approx \lnot 1\oplus \lnot 1\approx \lnot (\lnot 0)\oplus \lnot (\lnot 0)\approx 0\oplus 0\approx 0 \end{aligned}$$

are satisfied and hence $\mathbb A(\mathbb S(\mathbf A))=\mathbf A$.

If $\mathbf S=(A,|,1)$ is a strong Sheffer stroke NMV-algebra, $\mathbb A(\mathbf S)=(A,\oplus ,\lnot ,0)$ and $\mathbb S(\mathbb A(\mathbf S))=(A,|_1,1_1)$, then the identities

$$\begin{aligned}&x|_1y \approx \lnot x\oplus \lnot y\approx ((x|1)|1)|((y|1)|1)\approx x|y, \\&1_1 \approx \lnot 0\approx 0|1\approx 1|0\approx 1 \end{aligned}$$

are satisfied and hence $\mathbb S(\mathbb A(\mathbf S))=\mathbf S$. $\square$

We continue our investigations by considering implication NMV-algebras. Surprisingly, a Sheffer stroke can be introduced also in this case, but some of the axioms must be modified.

### Definition 4.5

A *weak Sheffer stroke NMV-algebra* is an algebra (*A*, |, 1) of type (2, 0) satisfying the identities

$$\begin{aligned}&(x|1)|1 \approx x, \\&x|(x|1) \approx 1, \\&x|(1|1) \approx 1, \\&1|x \approx x|1, \\&((x|1)|y)|y \approx ((y|1)|x)|x, \\&(x|1)|((y|x)|1) \approx 1, \\&x|(((((x|y)|y)|z)|z)|1) \approx 1, \\&(1|1)|x \approx 1. \end{aligned}$$

The operation | will be called the *weak Sheffer stroke*.

Similarly as before, the weak Sheffer stroke NMV-algebra can be derived by means of an implication NMV-algebra as follows.

### Theorem 4.6

Let $\mathbf A=(A,\rightarrow ,0)$ be an implication NMV-algebra with 0 and put

$$\begin{aligned} x|y:=x\rightarrow \lnot y \end{aligned}$$

for all $x,y\in A$. Then, $\mathbb W(\mathbf A):=(A,|,1)$ is a weak Sheffer stroke NMV-algebra.

### Proof

The following identities are satisfied:

$$\begin{aligned}&x|1 \approx x\rightarrow \lnot 1\approx x\rightarrow 0\approx \lnot x, \\&(x|1)|1 \approx \lnot (\lnot x)\approx x, \\&x|(x|1) \approx x\rightarrow \lnot (\lnot x)\approx x\rightarrow x\approx 1, \\&x|(1|1) \approx x\rightarrow \lnot (\lnot 1)\approx x\rightarrow 1\approx 1, \\&1|x \approx 1\rightarrow \lnot x\approx \lnot x\approx x\rightarrow 0 \\&\quad \approx x\rightarrow \lnot 1\approx x|1,\\&((x|1)|y)|y \approx (\lnot x\rightarrow \lnot y)\rightarrow \lnot y \\&\quad \approx (\lnot y\rightarrow \lnot x)\rightarrow \lnot x\approx ((y|1)|x)|x,\\&(x|1)|((y|x)|1) \approx \lnot x\rightarrow \lnot (\lnot (y\rightarrow \lnot x))\\&\quad \approx \lnot x\rightarrow (y\rightarrow \lnot x)\approx 1, \\&x|(((((x|y)|y)|z)|z)|1) \approx x\rightarrow \lnot (\lnot ((((x\rightarrow \lnot y)\rightarrow \lnot y)\\&\quad \rightarrow \lnot z)\rightarrow \lnot z)) \\&\quad \approx x\rightarrow ((((x\rightarrow \lnot y)\rightarrow \lnot y)\rightarrow \lnot z)\rightarrow \lnot z)\approx 1, \\&(1|1)|x \approx (1\rightarrow \lnot 1)\rightarrow \lnot x\approx \lnot 1\rightarrow \lnot x\approx 0\rightarrow \lnot x\approx 1. \end{aligned}$$

$$\square $$

Analogously, as it was the case for the strong Sheffer stroke, the weak Sheffer operation generates the fundamental operations of an implication NMV-algebra with 0.

### Theorem 4.7

Let $\mathbf S=(A,|,1)$ be a weak Sheffer stroke NMV-algebra and put

$$\begin{aligned}&x\rightarrow y := x|(y|1), \\&0 := 1|1 \end{aligned}$$

for all $x,y\in A$. Then, $\mathbb I_1(\mathbf S):=(A,\rightarrow ,0)$ is an implication NMV-algebra with 0.

### Proof

The following identities are satisfied:

- (8) $$x\rightarrow x\approx x|(x|1)\approx 1\approx y|(y|1)\approx y\rightarrow y$$,
- (9) $$x\rightarrow 1\approx x|(1|1)\approx 1$$,
- (10) $$1\rightarrow x\approx 1|(1|x)\approx (x|1)|1\approx x$$,
- (11) $$(x\rightarrow y)\rightarrow y\approx (x|(y|1))|(y|1)\approx (((x|1)|1)|(y|1))|(y|1)\approx (((y|1)|1)|(x|1))|(x|1)\approx (y|(x|1))|(x|1)\approx (y\rightarrow x)\rightarrow x$$,
- (12) $$x\rightarrow (y\rightarrow x)\approx x|((y|(x|1))|1)\approx ((x|1)|1)|((y|(x|1))|1)\approx 1$$,
- (13) $$x\rightarrow ((((x\rightarrow y)\rightarrow y)\rightarrow z)\rightarrow z)\approx x|(((((x|(y|1))|(y|1))|(z|1))|(z|1))|1)\approx 1$$,
- (14) $$0\rightarrow x\approx (1|1)|(x|1)\approx 1$$. $$\square $$

Again we can prove that the correspondence described by the last two theorems is one to one.

### Theorem 4.8

The above-mentioned correspondence is one to one.

### Proof

If $\mathbf A=(A,\rightarrow ,0)$ is an implication NMV-algebra with 0, $\mathbb W(\mathbf A)=(A,|,1)$ and $\mathbb I_1(\mathbb W(\mathbf A))=(A,\rightarrow _1,0_1)$, then the identities

$$\begin{aligned}&x\rightarrow _1y \approx x|(y|1)\approx x\rightarrow \lnot (y\rightarrow \lnot 1) \\&\quad \approx x\rightarrow \lnot (y\rightarrow 0)\approx x\rightarrow \lnot (\lnot y)\approx x\rightarrow y,\\&0_1 \approx 1|1\approx 1\rightarrow \lnot 1\approx 1\rightarrow 0\approx \lnot 1\approx 0 \end{aligned}$$

are satisfied and hence $\mathbb I_1(\mathbb W(\mathbf A))=\mathbf A$.

If $\mathbf S=(A,|,1)$ is a weak Sheffer stroke NMV-algebra, $\mathbb I_1(\mathbf S)=(A,\rightarrow ,0)$ and $\mathbb W(\mathbb I_1(\mathbf S))=(A,|_1,1_1)$, then the identities

$$\begin{aligned}&x|_1y \approx x\rightarrow \lnot y\approx x|((y|((1|1)|1))|1)\approx x|((y|1)|1)\approx x|y, \\&1_1 \approx \lnot 0\approx 0|(0|1)\approx 1 \end{aligned}$$

are satisfied and hence $\mathbb W(\mathbb I_1(\mathbf S))=\mathbf S$. $\square$

Let us note, finally, that the weak Sheffer stroke not only determines the operation $\rightarrow$ of the induced implication NMV-algebra with 0, but in fact also the induced poset as well as the induced directoid since we have $x\le y$ if and only if $x|(y|1)=1$ and, moreover, the identity $x\sqcup y\approx (x|(y|1))|(y|1)$ is satisfied.
